# Hepatoprotective Activity of *Flourensia cernua* and Its Impact on Aerobic Gut Microbiota in a Valproic Acid-Induced Injury Model in Wistar Rats

**DOI:** 10.3390/cimb48030248

**Published:** 2026-02-26

**Authors:** Jorge Martín Llaca-Díaz, José Miguel de la Rosa-García, Rocío Castro-Ríos, Diana Patricia Moreno-Pena, Marsela Garza-Tapia, Liliana Torres-Gonzalez, Lorena Salazar-Cavazos, Humberto Rodríguez-Rocha, Aracely García-García, Catalina Leos-Rivas, Verónica Mayela Rivas-Galindo, Luis Alejandro Pérez-López, Diana Raquel Rodríguez-Rodríguez, Linda E. Muñoz-Espinosa, Paula Cordero Pérez

**Affiliations:** 1 Department of Clinical Pathology, University Hospital Dr. José E. González, Universidad Autónoma de Nuevo León, Monterrey 64460, Nuevo León, Mexico; jorge.llacadz@uanl.edu.mx (J.M.L.-D.);; 2 Liver Unit, Department of Internal Medicine, University Hospital Dr. José E. González, Universidad Autónoma de Nuevo León, Monterrey 64460, Nuevo León, Mexico; jose.delarosaga@uanl.edu.mx (J.M.d.l.R.-G.); diana.morenope@uanl.mx (D.P.M.-P.); liliana.torresgzz@uanl.edu.mx (L.T.-G.); diana.rodriguezrgz@uanl.edu.mx (D.R.R.-R.); linda.munozesp@uanl.edu.mx (L.E.M.-E.); 3 Department of Analytical Chemistry, School of Medicine, Universidad Autónoma de Nuevo León, Monterrey 64460, Nuevo León, Mexico; rocio.castrors@uanl.edu.mx (R.C.-R.); marsela.garzatp@uanl.edu.mx (M.G.-T.); veronica.rivasgl@uanl.edu.mx (V.M.R.-G.); luis.perezlp@uanl.edu.mx (L.A.P.-L.); 4 Department of Histology, School of Medicine, Universidad Autónoma de Nuevo León, Monterrey 64460, Nuevo León, Mexico; humberto.rodriguezrc@uanl.edu.mx (H.R.-R.); aracely.garciagr@uanl.edu.mx (A.G.-G.); 5 Department of Chemistry, Faculty of Biological Sciences, Universidad Autónoma de Nuevo León, Monterrey 64460, Nuevo León, Mexico; catalina.leosrs@uanl.edu.mx

**Keywords:** *Flourensia cernua*, hepatoprotective, microbiota, liver, oxidative stress, valproic acid-model

## Abstract

Liver disease represents a major global health issue, with limited availability of effective hepatoprotective treatments. Hojasé or hojasén or *Flourensia cernua* (Fc) is known for its antioxidant properties and high phenolic content and may exhibit a potential hepatoprotective effect. Additionally, natural products have been shown to restore gut microbiota and reduce liver inflammation. To evaluate the hepatoprotective activity of Fc and its impact on gut microbiota in a valproic acid (VPA)-induced injury model in Wistar rats. Seven groups of Wistar rats (*n* = 6) were treated as follows: Sham; Non-toxic Fc (NTox 200 and 400 mg/kg); VPA 500 mg/kg; VPA + Fc 200 and VPA + Fc 400; and silybinin 500 mg/kg (VPA + Slb). Liver function tests, malondialdehyde (MDA) and superoxide dismutase (SOD) markers, aerobic gut microbiota analysis, and histological analysis were conducted. No significant differences were observed in ALT and AST levels between NTox 200, NTox 400, and Sham. Only the 400 mg/kg dose of Fc significantly reduced ALT and AST versus VPA, similar to Slb. In VPA + Fc 200 and VPA + Fc 400, MDA and SOD decreased versus VPA, comparable to VPA + Slb. Only VPA + Fc 400 and VPA + Slb restored aerobic gut microbiota versus VPA. No histological changes were observed between groups. Fc extract demonstrated hepatoprotective effects at a dose of 400 mg/kg and impacted the restoration of aerobic gut microbiota against VPA-induced damage.

## 1. Introduction

Liver disease accounts for more than 2 million deaths per year and is responsible for 4% of all deaths worldwide [1]. In Mexico, it ranks as the fourth leading cause of death, according to the National Institute of Statistics, Geography, and Informatics (INEGI) during the period from January to June 2023 [2]. These deaths are largely attributable to complications of cirrhosis and the development of hepatocellular carcinoma (HCC). The most common causes of cirrhosis are related to viral hepatitis, alcoholic liver disease, non-alcoholic fatty liver disease (NAFLD), and drug-induced liver injury (DILI) [1,3,4,5,6]. Regardless of its etiology, this disease is characterized by prolonged parenchymal damage, activation of the inflammatory response, and consequent fibrogenesis due to excessive accumulation of extracellular matrix components caused by activated hepatic myofibroblasts [7,8]. According to the METAVIR scoring system, fibrosis stages F1 to F3 are considered reversible with appropriate treatment that eliminates the etiologic agent and restores liver architecture and function at early stages [9], whereas stage F4 is considered irreversible [10]. For this reason, it is essential to perform timely diagnosis and treatment in the early stages of fibrosis to achieve disease resolution and prevent progression to cirrhosis or the development of HCC.

In order to develop new diagnostic and treatment tools for liver diseases, there remains significant interest in using animal models that replicate the clinical, biochemical, and histological characteristics of human liver diseases. In recent years, there has been a considerable increase in the use of valproic acid (VPA) in rodent liver damage [11], whose mechanism of hepatotoxicity is attributed to oxidative stress and an imbalanced antioxidant activity, both of which play a key role in the oxidative damage of macromolecules [12]. Furthermore, it has been demonstrated that VPA interferes with the enzyme carnitine palmitoyl-transferase I, which is crucial in the β-oxidation of mitochondrial fatty acids, thereby inducing hepatotoxicity and weight gain in patients under treatment with this drug [13]. Consequently, various studies have established liver damage models using VPA, highlighting its effects mainly at the histological and biochemical levels [14,15]. Additionally, this damage model has been employed to evaluate the potential hepatoprotective effects of plant extracts in in vivo experimental models [16,17]. Compounds or extracts from natural products with antioxidant effects are known to help prevent and/or treat chronic diseases related to oxidative stress by eliminating the high levels of reactive oxygen species (ROS) produced and by increasing the levels of the body’s own antioxidant enzymes [18,19]. For this reason, various plants and natural products have been studied to demonstrate their potential hepatoprotective activity against liver damage models in animals [20,21,22].

*Flourensia cernua* (Fc), more commonly known as *hojasé* or *hojasén*, is a bitter-tasting shrub with a hops-like aroma that grows in the deserts of northern Mexico. This plant has been used in traditional Mexican medicine primarily for the treatment of various gastrointestinal diseases or complications, such as stomach pain, indigestion, diarrhea, and dysentery [23,24]. Additionally, its antifungal and bactericidal activities have been demonstrated [25,26,27]. Various studies have also highlighted the antioxidant activity of the hydroalcoholic extract of this plant through in vitro assays, reporting a high polyphenol content, including flavones (glycosylated apigenin and luteolin), flavanones, and flavonols [28,29,30,31]. This suggests that these compounds may be responsible for its antioxidant activity, making it a potential candidate for assessing its possible hepatoprotective effect.

On the other hand, gut microbiota (GM) can be defined as a diverse ecosystem of microorganisms residing in the digestive tract, primarily composed of bacteria [32]. In healthy individuals, it performs numerous functions that benefit the host, such as regulating nutrient metabolism, drug metabolism and absorption, and the production of bioactive molecules, among other things [33,34]. Composition of the GM is complex and abundant, with the dominant phyla in the human intestine and other species being *Firmicutes*, *Bacteroidetes*, and *Actinobacteria*, which together account for more than 90% of intestinal bacteria [35,36]. The proportion of these phyla can be influenced by various internal and external factors, the most common of which include medication use, diet, an individual’s physiological state, and the onset and progression of diseases [37]. Various studies have shown a shift in the composition of normal GM associated with the onset and development of liver diseases, characterized by a reduced diversity of beneficial species and an increased presence of undesirable pathogenic species. This imbalance leads to alterations in the intestinal barrier, making it more permeable to bacterial components such as lipopolysaccharides, which can pass into the liver via the intestinal blood vessels. When this flow is constant, it can overload the liver, causing metabolic and immune disturbances [38,39].

Both experimental and human models have demonstrated that the use of natural products represents a potential treatment for GM restoration due to their diverse mix of compounds with antioxidant and anti-inflammatory effects, such as polyphenols. These compounds promote the growth of beneficial bacteria that contribute to overall health and restore the composition of the intestinal barrier, thereby preventing the translocation of pathogenic components to the liver and reducing inflammation [40,41]. Therefore, the objective of the present study was to evaluate the hepatoprotective activity of Fc and its impact on aerobic GM in a VPA-induced liver damage model in Wistar rats.

## 2. Materials and Methods

### 2.1. Reagents

Valproic acid (Sigma-Aldrich, Saint Louis, MO, USA), Silibinin (Sigma-Aldrich), Saline solution, Microdacyn^®^ 60 Solution (OCULUS, Invekra, S.A.P.I. de C.V., Mexico, Reg. No. 0258C2014 SSA), ketamine (Anesket, PiSA Agropecuaria, S.A. de C.V., Mexico, Reg. SAGARPA Q7833-028), and xylazine (Sedaject, Vedilab S.A. de C.V., Mexico, Reg. SAGARPA Q-0088-122).

### 2.2. Plant Material and Extract

The *Fluorensia cernua* plant material used in this study was obtained from Pacalli Company, Guadalupe, Nuevo León, México. The hydroalcoholic extract of *F. cernua* was kindly provided by Dr. Catalina Leos (Faculty of Biological Sciences, Autonomous University of Nuevo León). It was obtained through static maceration using methanol 80% for a duration of 7 days. Subsequently, filtration was performed, followed by a rotavaporation process.

### 2.3. LC-MS Analysis

An Ultimate 3000 (Thermo Dionex, Sunnyvale, CA, USA) UHPLC system and an LCQ Fleet (Thermo Fisher Scientific, Waltham, MA, USA) mass spectrometer were used for the analysis of the *Fc* extract. Chromatographic separation was achieved using a Supelco Discovery HS F5 column (150 × 2.1 mm, 3 μm) at 45 °C, with formic acid (0.1% *v*/*v*) and acetonitrile as mobile phases at a flow rate of 200 µL/min. The elution program started with 10% acetronitile for 1 min, increased to 70% for 19 min, and held for 20 min. After this, the mobile phase was returned to the initial composition, with a condition time of 20 min. The injection volume was 5 µL, and the UV-VIS detector was set at 254 nm. MS analysis was performed using an electrospray ion source (ESI) with nitrogen as sheath gas. The source was operated in the negative ion mode at 350 °C, a sheath gas flow of 40 U, sweep gas of 20 U, source voltage of 5 kV, capillary voltage of −22 V and tube lens voltage of −119.95 V. Data acquisition for MS and MS/MS analysis was made in the full scan mode from 50 to 2000 *m*/*z*, ion fragmentation was achieved using a normalized collision energy of 30%.

### 2.4. Animals

An experimental, prospective, and cross-sectional study was conducted using 7 groups of healthy Wistar rats with no prior procedures and no exclusions. (n = 6 in each group, of both sexes, and weighing 200–300 g). The rats were housed in polycarbonate cages under appropriate temperature and humidity conditions, with a 12 h light-dark cycle, free access to water, and standard rodent food (Nutricubo, Nutrimix S.A de C.V., Mexico). All animal procedures were carried out in accordance with proper laboratory animal care and use, as approved by the Ethics Committee of our institution (registration number QA24-00001) and in compliance with the specifications of the Mexican Official Standard NOM-062-ZOO-1999.

The sample size was decided based on the result of an a priori calculation using Equation (1):

(1) $$n=\frac{{\left(Z\alpha +Z\beta \right)}^{2}(\sigma_{1}^{2}+\sigma_{2}^{2})}{{\left(\mu_{1}-\mu_{2}\right)}^{2}}$$

Equation (1). Estimation of the sample size for comparison of means. Zα = Z-value for α; Zβ = Z-value for β; σ_1_ = expected standard deviation of group 1; σ_2_ = expected standard deviation of group 2; µ_1_ = expected mean of group 1; µ_2_ = expected mean of group 2.

#### Study Groups

Rats were randomized and divided into the following groups:

1. Healthy Control Group (Sham): Received 1 mL/day of 0.9% NaCl intraperitoneally (i.p.) for 7 days. Subsequently, for three days, 1 mL/day of 0.9% NaCl was administered via orogastric tube.
2. Non-Toxic Fc Control Groups (NTox 200 and 400): Received 1 mL/day of 0.9% NaCl i.p. for 7 days. Subsequently, for three days, 200 mg/kg/day or 400 mg/kg/day of a hydroalcoholic Fc extract was administered via orogastric tube, with a maximum application volume of 1 mL.
3. Valproic Acid (AVP) Damage Control Group: Received AVP at a dose of 500 mg/kg/day i.p. for 7 days. Subsequently, for three days, 1 mL/day of 0.9% NaCl was administered via orogastric tube.
4. Groups to Assess Hepatoprotective Activity of Fc (AVP + Fc 200 and AVP + Fc 400): Received AVP at a dose of 500 mg/kg/day i.p. for 7 days. Subsequently, for three days, 200 mg/kg/day or 400 mg/kg/day of a hydroalcoholic Fc extract was administered via orogastric tube with a maximum application volume of 1 mL.
5. Positive Hepatoprotection Control Group (AVP + Slb): Received AVP at a dose of 500 mg/kg/day i.p. for 7 days. Subsequently, 200 mg/kg/day of silibinin was administered via orogastric tube with a maximum application volume of 1 mL.

After 10 days of administration, all animals were anesthetized i.p. with 10 mg/kg of xylazine (Sedaject, Vedilab S.A. de C.V., Ciudad de México, Mexico, Reg. SAGARPA Q-0088-122) and 100 mg/kg of ketamine (Anesket, PiSA Agropecuaria, S.A. de C.V., Ciudad de Mexico, Mexico, Reg. SAGARPA Q7833-028) and underwent laparotomy on a surgical table. An abdominal incision was made to locate, dissect, and extract the liver. Following this, blood samples were obtained by exsanguination via puncture of the abdominal artery to collect a maximum volume of 5 mL. Afterward, euthanasia was performed in accordance with the NOM-062-ZOO-1999 recommendations. Blood samples were centrifuged for 15 min at 3500 rpm, and the serum was separated into aliquots and stored at −70 °C until subsequent analysis. The liver tissue was divided into two fragments: one for histopathological analysis, which was placed in 10% formalin, and the other for oxidative stress marker determination, which was frozen at −70 °C in cryovials for further analysis.

The investigators were blinded during outcome assessment and data analysis to ensure objectivity and minimize bias.

### 2.5. Biochemical Analysis

Serum samples were analyzed using an ILab Aries system (Instrumentation Laboratory, Werfen Group, Barcelona, Spain). Biochemical markers were evaluated, including albumin (ALB), alanine aminotransferase (ALT), alkaline phosphatase (ALP), aspartate aminotransferase (AST), lactate dehydrogenase (LDH), total proteins (TP), and glucose, using commercial kits according to the manufacturer’s instructions.

### 2.6. Determination of Oxidative Stress Markers

200 mg of liver tissue was weighed and mixed with 1000 µL of Tris-EDTA-dithiothreitol, then homogenized until a uniform appearance was obtained. The homogenate was centrifuged, and the supernatant was separated. Commercial kits (Cayman Chemical Company, Ann Arbor, MI, USA) were used for the determination of superoxide dismutase (SOD) and malondialdehyde (MDA) in the tissue homogenates, following the manufacturer’s instructions for each determination. Absorbance was measured according to the specifications of each kit using a microplate reader (Thermo Fisher Scientific, Carlsbad, CA, USA). Results were normalized using the Bradford method (Kruger NJ).

### 2.7. Histological Analysis

Histological analysis was conducted on previously collected liver tissue stored in histocassettes and preserved in 10% formaldehyde buffered at pH 7.4. Liver sections were prepared, and hematoxylin–eosin staining was performed. Stained histological sections were observed under an optical microscope (10× and 40×) to identify morphological changes. Morphometric analysis was performed at 40× magnification using the ImageJ program (version 1.54r). The morphometric parameter quantified was the number of binucleated cells in the centrilobular area (adjacent fields of the central vein) and the periportal area (adjacent fields of the portal triad) across 5 random fields in each region, calculating the percentage per field [42]. Digital images were captured with a Nikon Eclipse 50i microscope (Nikon Instruments Inc., Melville, NY, USA) and Q-capture Pro 7 software.

### 2.8. Microbiota Analysis

For the analysis of aerobic microbiota (MI), fecal samples were collected from each animal in the different study groups both pre-treatment and post-treatment. The analysis was carried out in two stages.

Sample Preparation and Colony Counting: From the collected fecal samples, the exact weight was taken, and 1 mL of sterile 0.9% NaCl was added and thoroughly mixed. Then, calibrated inoculation loops of 1 μL and 10 μL were used to inoculate the following culture media: eosin methylene blue (EMB), thiosulfate-citrate-bile salts-sucrose, MacConkey, and xylose-lysine-desoxycholate (XLD). The plates were incubated under aerobic conditions at 37 °C for 72 h. Upon colony growth, each distinct colony morphology was counted, and results were expressed as colony-forming units per gram (CFU/g).

MALDI-TOF Spectroscopy Analysis: Once bacterial colonies exhibited growth, distinct colonies were identified and isolated on a new EMB plate. Each colony was then applied to the Microflex LT MALDI-TOF plate and overlaid with α-cyano-4-hydroxycinnamic acid matrix, which was allowed to dry. The prepared plate was introduced into the BD Bruker Maldi Biotyper system, which obtained mass spectra of the identified bacteria. The spectra were compared against the system’s database to determine bacterial identity. A score of 2.0 or higher indicated species-level identification with high confidence, scores between 1.7 and 1.9 suggested genus-level identification, and scores below 1.7 did not warrant identity assignment.

After bacterial identification, the data were correlated with their respective morphologies and CFU/g for each colony. The total bacterial load was calculated by summing the CFU/g across all rats in each study group.

### 2.9. Statistical Analysis

A Shapiro–Wilk test was performed to assess the normality of the data. The results are expressed as mean ± standard deviation (SD) and median with interquartile range. For parametric data, group comparisons were performed using one-way analysis of variance (ANOVA) followed by Tukey’s post hoc test, utilizing Prism software (v. 8.0; GraphPad, San Diego, CA, USA). A *p*-value < 0.05 was considered statistically significant.

## 3. Results

### 3.1. LC-MS Analysis

**Figure 1:**
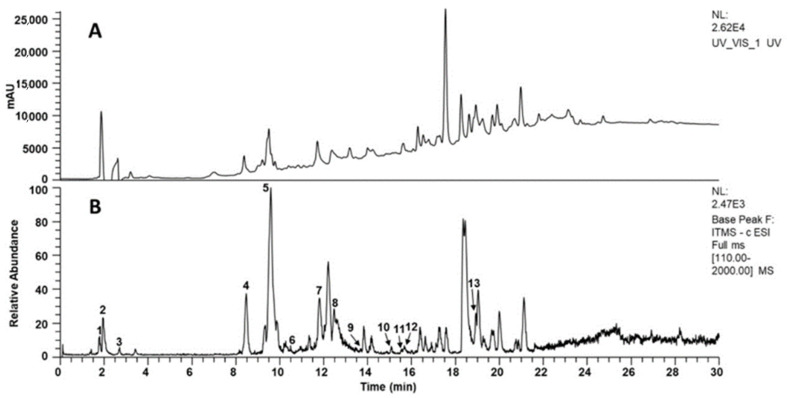
Representative chromatograms obtained from Flourensia extract (10 mg/mL). (**A**) HPLC-UV-Vis at 254 nm; (**B**) ESI-MS base peak chromatogram (negative ion mode, *m*/*z* 50–2000). Peaks are numbered and tentatively identified as: 1 = 4-O-feruloylquinic acid, 2 = 4-caffeoylquinic acid, 3 = genistein, 4 = vincenin-2, 5 = apigenin-6-glucoside-8-arabinoside, 6 = quercetin-3-O-rhamnohexoside, 7 = 1,5-dicaffeoylquinic acid, 8 = 1,4-dicaffeoylquinic acid, 9 = 3-O-caffeoyl-4-O-feruloylquinic acid, 10 = kaempferol isomer, 11 = quercetin, 12 = quercetin derivative, 13 = quercetin 3,7-dimethyl ether.

The extract was analyzed by LC-MS in both positive and negative ion modes, but the highest number of signals was observed in negative mode. Peak monitoring with the UV detector was performed at 210, 254, 280, and 310 nm, with the highest number of chromatographic peaks observed at 254 nm. Figure 1 shows the chromatograms obtained. LC-MS analysis allowed the identification of some of the secondary metabolites in the extract, including chlorogenic acids and flavonoids. Table 1 presents the data obtained for the identification of these compounds.

### 3.2. Evaluation of Non-Toxicity and Hepatoprotective Activity of the Hydroalcoholic Extract of Flourensia cernua by Measuring Biochemical Markers

**Figure 2:**
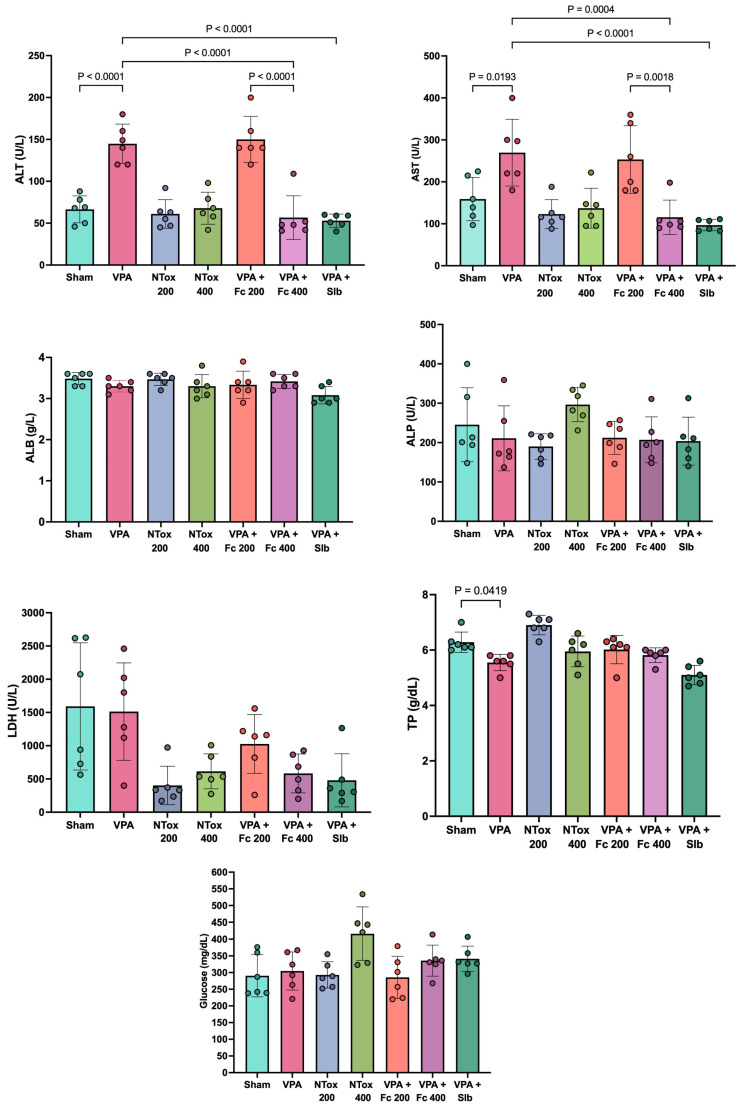
Biochemical markers in the different study groups. ALT: alanine aminotransferase; AST: aspartate aminotransferase; ALB: albumin; ALP: alkaline phosphatase; LDH: lactate dehydrogenase; U/L: units per liter; Sham: healthy control; VPA: valproic acid; NTox 200: non-toxicity 200 mg/kg; NTox 400: non-toxicity 400 mg/kg; VPA + Fc 200: treatment 200 mg/kg; VPA + Fc 400: treatment 400 mg/kg; VPA + Slb: positive control 200 mg/kg. Analysis by ANOVA with post hoc Tukey test. Values expressed as mean ± standard deviation.

Serum concentrations of ALT, AST, ALB, ALP, LDH, total protein, and glucose were analyzed across the different study groups. The liver damage model induced by AVP showed a significant increase in ALT and AST levels in the AVP group (144.8 ± 23.4 U/L and 269.5 ± 79.5 U/L, respectively) compared with the Sham group (66.5 ± 16.1 U/L and 159.0 ± 51.6 U/L, respectively). Additionally, a significant decrease in total protein was observed in the AVP group (5.6 ± 0.3 g/L) compared with the Sham group (6.3 ± 0.4 g/L). The remaining biochemical markers, as well as the evaluated histology, showed no significant change between the two groups (Figure 2).

When analyzing the non-toxic effects of the extract at the administered doses, no significant difference in ALT and AST was observed between the NTox 200 group (61.0 ± 17.1 U/L and 123.2 ± 34.3 U/L, respectively) and the NTox 400 group (67.8 ± 19.2 U/L and 137.2 ± 47.4 U/L, respectively) compared with the Sham group (66.5 ± 16.1 U/L and 159.0 ± 51.6 U/L).

In evaluating the hepatoprotective effect of *Flourensia cernua* (Fc), it was observed that the AVP + Fc 200 group showed no significant difference in ALT (150.0 ± 27.6 U/L) and AST (253.3 ± 80.7 U/L) compared with the AVP group (ALT: 144.8 ± 23.4 U/L and AST: 269.5 ± 79.5 U/L). However, the AVP + Fc 400 group showed a significant decrease in ALT (56.7 ± 25.9 U/L) and AST (115.5 ± 41.0 U/L) compared with the AVP group, showing similar results to the AVP + Slb group, where ALT (53.0 ± 7.9 U/L) and AST (96.8 ± 13.5 U/L) showed no significant differences between the two groups (Figure 2).

**Figure 3:**
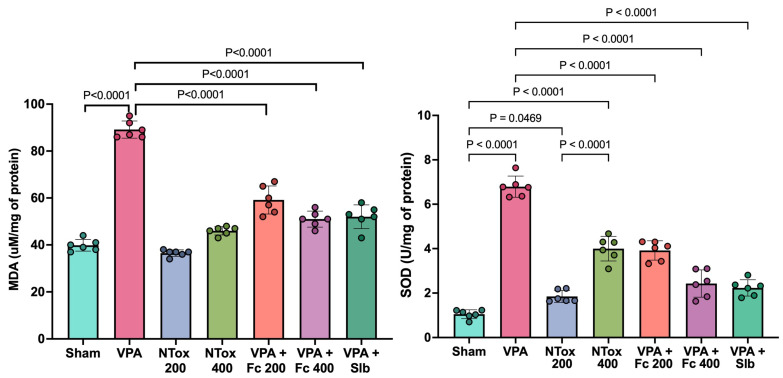
Oxidative stress markers across the different study groups. MDA: malondialdehyde; SOD: superoxide dismutase; Sham: healthy control; VPA: valproic acid; NTox 200: non-toxicity 200 mg/kg; NTox 400: non-toxicity 400 mg/kg; VPA + Fc 200: treatment 200 mg/kg; VPA + Fc 400: treatment 400 mg/kg; VPA + Slb: positive control 200 mg/kg. Analysis by ANOVA with post hoc Tukey test. Values expressed as mean ± standard deviation.

The levels of MDA and SOD were analyzed across the different study groups. Both markers showed a significant increase in the AVP group (89.6 ± 3.5 μM/mg and 6.7 ± 0.5 U/mg) compared with Sham (39.8 ± 2.5 μM/mg and 1.1 ± 0.2 U/mg). For MDA, NTox 200 (36.5 ± 1.4 μM/mg) and NTox 400 (46.0 ± 1.8 μM/mg) showed no significant differences compared with Sham. However, for SOD, there was a significant increase in activity in NTox 200 (1.9 ± 0.3 U/mg) and NTox 400 (3.9 ± 0.6 U/mg) compared with Sham (Figure 3).

### 3.3. Evaluation of Non-Toxicity and Hepatoprotective Activity of the Hydroalcoholic Extract of Flourensia cernua Through Morphological Assessment of Study Groups

**Figure 4:**
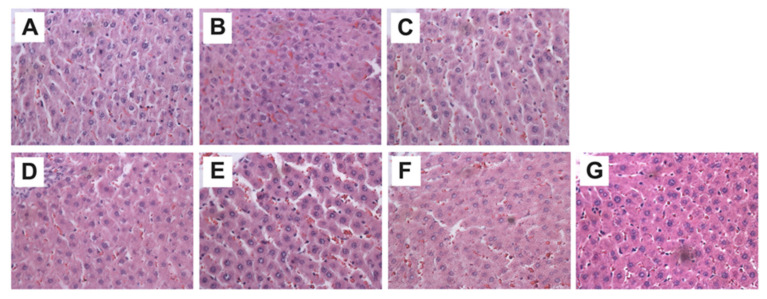
Representative hepatic micrographs of experimental groups. Hematoxylin and eosin staining (40×). (**A**) Sham: healthy control; (**B**) VPA: valproic acid; (**C**) NTox 200: non-toxicity 200 mg/kg; (**D**) NTox 400: non-toxicity 400 mg/kg; (**E**) VPA + Fc 200: treatment 200 mg/kg; (**F**) VPA + Fc 400: treatment 400 mg/kg; (**G**) VPA + Slb: positive control 200 mg/kg.

Histological analysis was performed across the different study groups using H&E staining, showing no significant structural changes in the treated groups compared with Sham (Figure 4).

**Figure 5:**
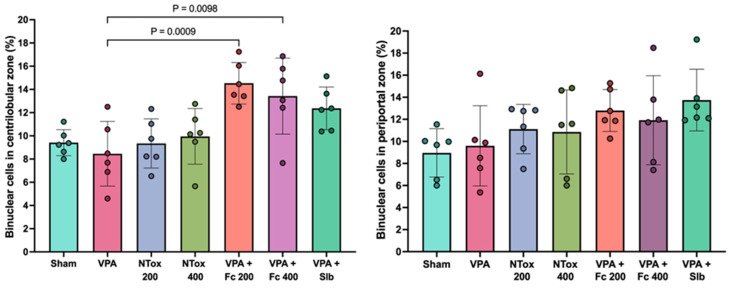
Comparison of the percentage of hepatocyte binucleation abundance across two distinct regions in the different study groups. Sham: healthy control; VPA: valproic acid; NTox 200: non-toxicity 200 mg/kg; NTox 400: non-toxicity 400 mg/kg; VPA + Fc 200: treatment 200 mg/kg; VPA + Fc 400: treatment 400 mg/kg; VPA + Slb: positive control 200 mg/kg.

Additionally, binucleated cell counts were conducted in two specific regions: the centrolobular and periportal zones. No significant difference was observed between AVP and SHAM groups; however, a significant increase in the abundance of binucleated cells was noted in the centrolobular region in both F. cernua treated groups compared with VPA (Figure 5).

### 3.4. Aerobic Intestinal Microbiota Using MALDI-TOF

**Figure 6:**
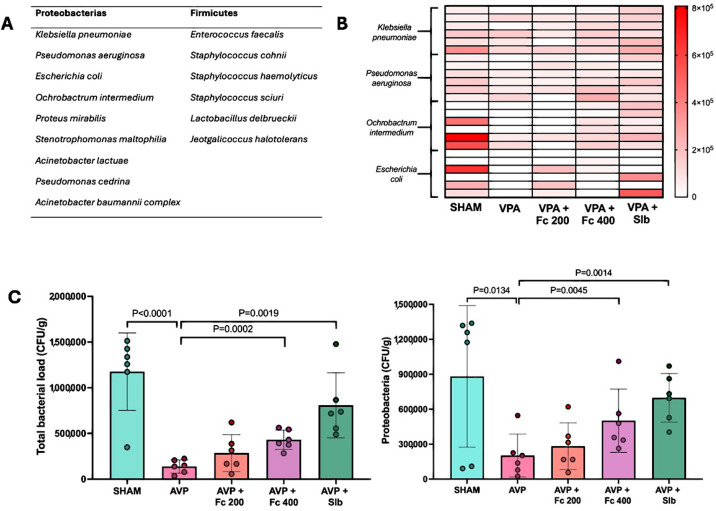
(**A**) Bacterial species identified with high reliability in the feces of the study groups by MALDI-TOF. (**B**) Heat map of total bacterial counts across the different study groups. (**C**) Total bacterial load and Proteobacteria in the various study groups. CFU/g: colony-forming units per gram; Sham: healthy control; AVP: valproic acid; AVP + Fc 200: treatment 200 mg/kg; AVP + Fc 400: treatment 400 mg/kg; AVP + Slb: positive control 200 mg/kg. Values are expressed as median ± interquartile range.

In the evaluation of MI across the various study groups, the identification of 15 distinct bacterial species with high-confidence identification scores was performed. Among these, *Klebsiella pneumoniae*, *Pseudomonas aeruginosa*, *Escherichia coli*, and *Ochrobactrum intermedium* were the most frequently observed in all study groups (Figure 6).

Based on the most frequently identified bacteria, a heat map was created to observe the intensity related to CFU/g across the different study groups. It was observed that AVP exhibited the lowest intensities, while AVP+200 and AVP+400 showed partial restoration in the counts of these bacteria, with this effect being more pronounced in AVP + Slb (Figure 6).

When comparing total bacterial load, it was observed that AVP exhibited a significant reduction compared with SHAM. On the other hand, only the AVP + Fc 400 group showed a significant increase in bacterial load and Proteobacteria levels, similar to AVP + Slb. No significant difference was observed between AVP + Fc 400 and AVP + Slb in these parameters (Figure 6).

## 4. Discussion

The chemical profiling revealed a predominance of phenolic compounds, mainly chlorogenic acids and flavonoids, in agreement with previous reports for the family *Asteraceae* and the genus *Flourensia* [31,43]. Among the identified phenolic acids, mono- and dicaffeoylquinates, as well as feruloylquinates, were prominent, indicating an active phenylpropanoid biosynthetic pathway. The detected flavonoids were mainly glycosylated flavones and flavonols derived from apigenin, quercetin, and kaempferol, including compounds such as vicenin-2 and apigenin-6-glucoside-8-arabinoside, which have been associated with structural stability, antioxidant activity, and photoprotective functions. Flavonoids reported in other studies, such as myricetin or rhoifolin, as well as anthocyanins, were not detected [43], suggesting a chemical profile dominated by non-pigmented phenolic compounds.

A noteworthy finding was the presence of genistein, an isoflavonoid not previously reported in this genus, which may reflect chemotaxonomic variability or specific stress-related or developmental conditions. In contrast to studies focused on lipophilic metabolites with phytotoxic activity [43], the profile described here is characterized by polar polyphenols, indicating a metabolic orientation toward antioxidant functions and general defense mechanisms. Overall, these results expand the current phytochemical knowledge of the species by confirming the predominance of glycosylated flavonoids and chlorogenic acid derivatives, while also incorporating previously unreported compounds, thereby reinforcing the relevance of this study for the phytochemical and chemotaxonomic characterization of the analyzed material.

The use of animal experimental models that reflect liver damage represents the clinical characteristics associated with liver disease in humans and allows the development of new treatment tools. AVP has been widely used to induce acute liver damage, reporting an increase in ALT and AST, which indicates cell lysis [14,44]. A significant increase in these enzymes has been reported when using AVP at a dose of 500 mg/kg twice daily for 7 days [45], which aligns with the present study, where a significant increase in ALT and AST was observed when comparing AVP versus SHAM. On the other hand, it is known that a decrease in total protein is indicative of defective protein biosynthesis. This has been reported in carbon tetrachloride poisoning, another inducer of liver damage [46], as well as with AVP at the hepatic tissue level [47]. This was similar to what was observed in the present study, where a significant decrease in PT was found in AVP compared with SHAM.

In the present study, the hepatoprotective effect of the methanolic extract from the aerial part of *Flourensia cernua* was evaluated. We found that the extract at doses of 200 and 400 mg/kg did not show toxic effects, as no significant differences were observed between NTox 200 and NTox 400 when compared with SHAM. This finding is consistent with other studies on this plant [31,48], which report similar findings; dose comparisons should be taken into account for contrasting the results. When evaluating the extract’s effect, it was observed that in VPA + Fc 200, the extract did not show a hepatoprotective effect against AVP. However, in VPA + Fc 400, this effect was observed, as evidenced by a significant reduction in AST and ALT.

On the other hand, oxidative stress is described as being caused by an imbalance between the generation of reactive oxidants and the neutralization capacity of endogenous antioxidant systems. This imbalance leads to the production of reactive species, resulting in damage [49]. Lipid peroxidation is considered a process in which free radicals attack lipids, leading to the formation of products that include lipid peroxyl radicals and hydroperoxides, with MDA being one of the main secondary products [50]. MDA has been reported in various studies as significantly increased with the use of AVP in acute damage models [51,52]. These results are similar to those observed in the present study, where a significant increase in MDA was found in AVP compared with SHAM. Additionally, it was observed that both groups treated with *F. cernua* extract and the silibinin control group showed significantly lower levels of hepatic MDA content compared with AVP.

On the other hand, SOD is considered the main cellular mechanism for combating oxidative stress. In the present study, a significant increase in SOD was observed in both NTox groups in a dose-dependent manner. These findings are comparable to other studies where the oral administration of hydroalcoholic extracts from various plants alone increased enzyme activity in a dose-dependent manner [53,54]. This suggests that the extract of *F. cernua* possesses in vivo antioxidant activity and is capable of mitigating the effects of ROS in biological systems. Moreover, it has been reported that plants contain various SOD isoforms, which play an important role in stress tolerance [55]. It can be inferred that the observed increase in SOD activity is intrinsic to the plant’s existing contents and not due to toxicity. This is considered a protective mechanism mediated by the extract.

A significant increase in SOD has been reported in acute damage models with AVP. This can be explained as a compensatory mechanism to counteract the oxidative stress associated with hepatotoxicity [45]. This finding aligns with the present study, where a significant increase in SOD activity was observed in AVP compared with SHAM. Additionally, it was observed that the groups treated with *F. cernua* extract restored SOD activity. Notably, the *F. cernua* 400 group exhibited the same activity as AVP + Slb, suggesting its potential antioxidant effect.

The histological analysis results did not show significant differences between the treated groups compared with the SHAM group in both Fc200 and Fc400 groups, as well as in groups treated with AVP. These findings may be attributed to the exposure time and/or duration of the various treatments with AVP not being sufficient to reflect changes at this level. It has been reported that AVP is a liver damage-inducing agent at prolonged exposure times, evident through hepatic sinusoidal dilation, congestion, and inflammatory cell infiltration [44]. In the present study, no significant differences were observed between the AVP group and the SHAM group in the number of binucleated cells, even though a lower number of these cells would have been expected in the AVP group due to induced hepatotoxicity. Binucleated cell counts are significant because during liver regeneration after damage, hepatocytes undergo one or two rounds of division to restore lost hepatic mass [56]. However, a significant increase in the number of binucleated cells was observed in the centrolobular area in both VPA + Fc 200 and VPA + Fc 400 groups. This suggests that the hydroalcoholic extract of this plant induces hepatic division, which could indicate a protective mechanism at this level.

The composition of the MI can be influenced by various internal and external factors, with one of the most common being the use of medications, such as those used to treat seizures like AVP. It has been reported that its use in patients affects the composition and diversity of the MI [57]. Although the role of AVP on the MI has not been proven in liver damage models, other authors have reported that AVP-induced damage causes intestinal bacterial dysbiosis [36]. This is consistent with the present study, where the AVP group showed a considerable reduction in total bacterial load compared with the SHAM group. Moreover, it has been shown that natural products are a potential treatment for restoring the MI, mainly due to the antioxidant and anti-inflammatory effects of polyphenols [40,41]. These observations align with the findings of the present study, where groups treated with AVP + Fc at various doses showed restoration of normal MI levels compared with the AVP group, an effect that was also observed with silibinin.

## 5. Conclusions

The hepatoprotective effect of *Flourensia cernua* extract at a dose of 400 mg/kg was demonstrated, as well as its impact on the restoration of the aerobic MI against damage induced by AVP at this dose. The regulatory pathways of the hepatoprotective activity and the isolation of the molecules responsible for this effect remain to be established. These findings support its potential practical application as a nutraceutical supplement or adjuvant therapy for the prevention or management of drug-induced liver injury, pending further clinical and long-term safety studies.

## Figures and Tables

**Table 1:** Tentative assignment of compounds in the *Flourensia* extract based on LC-ESI-MS/MS analysis in negative ionization mode [43,44,45,46,47].

(No)	[M-H] (*m*/*z*)	Fragment Ions (*m*/*z*)	Identified Compounds
1	367	173 (bp), 191	4-O-feruloylquinic acid
2	515	353 (bp), 191, 173, 135	4-caffeoylquinic acid
3	269	269 (bp), 225, 224, 201, 241, 240	Genistein
4	593	473 (bp), 353, 383, 503	Vincenin-2
5	563	443, 383, 353 (bp), 473, 545, 503	Apigenin-6-glucoside-8-arabinoside
6	609	179, 301 (bp), 463	Quercetin-3-*O* rhamno hexoside
7	515	353 (bp), 191, 173	1,5-dicaffeoylquinic acid
8	515	353 (bp), 191, 173, 317	1,4-dicaffeoylquinic acid
9	529	367 (bp), 335, 349	3-O-caffeoyl,4-O-feruloylquinic acid
10	285	285 (bp), 257, 175, 151	Kaempferol isomer
11	301	179 (bp), 151, 255, 273, 257	Quercetin
12	316	301 (bp), 300, 284	Quercetin derivative
13	329	314 (bp), 315, 313, 286, 299, 311,243	Quercetin 3,7-dimethyl ether

bp: base peak.

## Data Availability

The raw data supporting the conclusions of this article will be made available by the authors on request.

## Abbreviations

The following abbreviations are used in this manuscript

ALB	Albumin
ALP	Alkaline Phosphatase
ALT	Alanine Aminotransferase
AST	Aspartate Aminotransferase
FC	*Flourensia Cernua*
HCC	Hepatocellular Carcinoma
LDH	Lactate Dehydrogenase
MDA	Malondialdehyde
MI	Microbiota
NAFLD	Non-Alcoholic Fatty Liver Disease
ROS	Reactive Oxygen Species
SOD	Superoxide Dismutase
TP	Total Proteins
VPA	Valproic Acid
